# Ideal Cardiovascular Health and Cognitive Test Performance: Testing a Modified Index of Life's Simple 7 Among Older Chinese Adults

**DOI:** 10.3389/fpubh.2018.00352

**Published:** 2018-11-28

**Authors:** Theresa E. Gildner, Nawi Ng, Fan Wu, Yanfei Guo, J. Josh Snodgrass, Paul Kowal

**Affiliations:** ^1^Department of Anthropology, Dartmouth College, Hanover, NH, United States; ^2^Unit of Epidemiology and Global Health, Department of Public Health and Clinical Medicine, Umeå University, Umeå, Sweden; ^3^Centre for Demographic and Ageing Research, Faculty of Social Sciences, Umeå University, Umeå, Sweden; ^4^Shanghai Municipal Center for Disease Control and Prevention, Shanghai, China; ^5^World Health Organization SAGE, Geneva, Switzerland; ^6^Priority Research Centre for Generational Health and Ageing, University of Newcastle, Newcastle, NSW, Australia; ^7^Research Institute for Health Sciences, Chiang Mai University, Chiang Mai, Thailand

**Keywords:** cardiovascular disease risk factors, dementia prevention, global aging, lifestyle, heart disease

## Abstract

Evidence suggests that cognitive decline in older adults is influenced by cardiovascular health (CVH), with metabolic and vascular mechanisms hypothesized to underlie the etiology of cognitive impairment. Research in high-income nations suggests that improved CVH is linked with decreased cognitive impairment risk, but it is unclear if this pattern is evident in low-income countries. Nationally-representative data collected in China were drawn from the World Health Organization's Study on global AGing and adult health Wave 1 (2007–2010; *n* = 11,295). Seven CVH factors were classified as “ideal” or “not ideal”: smoking and drinking frequency, body mass index, physical activity level, blood pressure, diet, and self-reported anxiety. Additionally, scores from five cognitive performance tests (immediate and delayed verbal recall, forward and backward digit span, verbal fluency) were used to create a composite cognitive function variable. Linear regression analyses tested whether ideal CVH measures were associated with higher composite cognitive performance, controlling for sociodemographic factors. As hypothesized, ideal CVH was generally associated with higher cognitive performance. Low anxiety levels and reliable access to sufficient food (including produce) were particularly associated with higher cognitive function. These results suggest early detection and controlling modifiable CVH risks may protect aging individuals in China from cognitive decline.

## Introduction

The global population is currently experiencing a rapid aging transition, largely due to increased life expectancies and decreased fertility in many countries; however, global aging and health patterns are not uniform. At the start of the Twenty-first century, the number of Chinese adults aged 60 years and older reached an estimated 130 million, roughly 10% of the total Chinese population and 21% of the world's older population (1, 2). This trend is expected to continue such that the population of older adults in China will nearly triple and comprise 25% of the national population by 2030 (2), presenting considerable opportunities and challenges for China in coming decades. To put this into context, China had more cases of Alzheimer's disease in 2010 than any other country in the world (3). Over the last three decades, the prevalence of both Alzheimer's disease and vascular dementia has significantly increased, and it appears previous work has underestimated the burden and growth of dementia in China (4).

This increased disease burden will challenge healthcare systems to reform as they seek to meet the demands of patients and their caregivers in the coming years. It is therefore critical to identify potential interventions to promote healthy aging and reduce both the economic and social costs of dementia. Evidence suggests that improving cardiovascular health (CVH) among older adults represents one such prevention strategy. Factors associated with poor vascular health (such as smoking, obesity, low physical activity levels) are associated with cognitive aging (5, 6). Specifically, vascular and metabolic mechanisms may underlie the etiology of cognitive impairment, and poor vascular and metabolic health likely contribute to cognitive deficit at older ages (7). Models linking poor vascular/metabolic health to cognitive performance indicate that potential pathways between poor CVH and cognitive deficit include metabolic imbalance, altered distribution of cerebral blood flow, nerve demyelination, and microinfarction (8). Several factors that impair vascular and metabolic health have also been linked with increased dementia risk in older adults: smokers experience a significantly more pronounced decline in cognitive function, flexibility, and speed (9); high alcohol consumption has been associated with poorer neurological function (10); and, overweight and obese adults display significantly lower cognitive function compared to healthy weight individuals (8, 11).

Conversely, physical activity level (PAL) appears to be related to improved neuronal plasticity and the release of hormones linked with neuronal creation and function; physical inactivity may therefore exacerbate cognitive decline (12). High blood pressure also appears to be associated with an increased risk of cognitive decline, including neural atrophy and lesions (13). Thus, hypertension may contribute to dementia risk (13). Further, poor diet (including low fruit and vegetable intake and the inability to acquire sufficient food) is linked with diminished cognitive function via impaired metabolic and vascular health, increasing subsequent risk of diabetes and coronary heart disease (14). Finally, chronic psychological stress is associated with poor cardiovascular health, potentially leading to cognitive decline (15).

Prior studies have combined several of these CVH risk factors into a panel that represents overall CVH: Life's Simple 7, a proxy measure used to test the cumulative influence of CVH on cognitive performance in older persons (6). Previous findings support the hypothesis that individuals with healthier CVH measures are more resistant to impaired cognitive processing speed, executive function, and episodic memory (6). However, this work has focused primarily on populations from high-income nations. It is unclear whether these patterns are also evident in lower income nations. This research is especially important given the substantial increases in older adults residing in LMICs, such as China, expected to occur in coming years (2). It is therefore necessary to determine whether interventions designed to improve cognitive performance during aging through targeting CVH factors can be expected to have the same effects in all populations.

The present study addresses this need by examining the links between CVH factors and cognitive performance in China using data from the World Health Organization Study on global AGing and adult health (SAGE) Wave 1 (16). This research clarifies how a modified panel of seven factors known to influence CVH (smoking, alcohol consumption, body mass index [BMI; calculated from individual height and weight values], PAL, blood pressure, diet, and self-reported anxiety) is linked with specific measures of cognitive performance among older Chinese individuals, while controlling for sex, age, wealth, and education level. These results will help determine whether CVH is linked with poor cognitive function in China. These findings also have potentially important clinical applications, including the identification of CVH factors most strongly linked with cognitive deficits in this population. This knowledge is required for the development of interventions designed to successfully improve CVH and subsequently reduce dementia risk. We hypothesize that the combined ideal measure score of the seven CVH factors will be positively associated with performance on a battery of cognitive function tests.

## Materials and methods

### Study design and participants

A nationally-representative sample of adults ≥50 years old (*n* = 13,367) were collected using a multistage stratified cluster sample design (16, 17). In-person interviews were used to collect household and individual level data between 2007 and 2010. Specifically, eight of China's 22 provinces were randomly selected. One county in the rural regions and one district/city in the urban regions was then selected at random from each province. Next, four townships in each selected county and four community blocks in each selected city were selected using probability proportional to size (PPS) sampling, for a total of 64 primary sampling units. Two villages/enumeration areas per township/community were then selected using PPS sampling, for a total of 128 secondary sampling units. Two residential blocks were then selected from each village/neighborhood community using random cluster sampling, for a total of 256 tertiary sampling units. Finally, 84 households were randomly selected (with a 95% response rate) from each sampled residential block using simple random sampling. Post-stratification corrections were applied to these weights to compensate for under-coverage. All analyses are carried out using these weights. At the time of data collection, China was classified as a lower middle-income nation (18).

### Ethical approval

SAGE China was approved by the Chinese Center for Disease Control and Prevention Ethical Review Committee, and World Health Organization's Ethical Review Committee. Written informed consent was obtained from all study participants.

### Measures of cardiovascular health

To test associations between CVH and cognitive function, seven measures associated with CVH were included in the analyses: smoking, alcohol consumption, BMI, PAL, blood pressure, diet, and self-reported anxiety. Each of these factors was classified as either “ideal” or “not ideal.” This approach was largely based on criteria defined by the American Heart Association (6). First, ideal smoking was defined as never smoked (6). Second, ideal alcohol consumption was classified as do not drink daily (10). Third, ideal BMI was calculated from measured height and weight and was defined as 18.5–22.9 kg/m^2^, based on the modified WHO definition of normal, healthy BMI in Asian populations (19).

Fourth, ideal PAL was defined as ≥ 150 min/week (6). Total PAL was calculated from interview data using questions from the Global Physical Activity Questionnaire (GPAQ) to determine self-report physical activity patterns (20). This questionnaire collects information about physical activity patterns across a range of daily activities. Participants reported the amount of time during a typical day spent in vigorous-intensity activities (those that caused large increases in breathing or heart rate) as part of their work, in moderate-intensity activities (those that caused small increases in breathing or heart rate) as part of their work, in vigorous-intensity activities during leisure time, and in moderate-intensity activities during leisure time. Self-reported time spent in vigorous or moderate exercise for both work and leisure were combined to create a composite PAL measure (min/week).

Fifth, ideal overall blood pressure was defined as exhibiting both ideal SBP (< 120 mm Hg) and ideal DBP (< 80 mm Hg) (6). Participant systolic (SBP) and diastolic blood pressure (DBP) were individually measured three times using a Boso Medistar Wrist Blood Pressure Monitor Model S. The three SBP values were averaged together to create a composite SBP measure; likewise, the three DBP values were combined to create an average DBP measure. Sixth, ideal diet was defined as typically consuming ≥ 5 servings of fruit or vegetables daily combined with reporting access to an adequate amount of food (6). Finally, given the negative effects of chronic anxiety on CVH (15), ideal anxiety levels were defined as reporting either no or mild anxiety, conversely not ideal anxiety was defined as reporting moderate, severe, or extreme levels of anxiety. Specifically, respondents were asked: “Overall in the last 30 days, how much of a problem did you have with worry or anxiety?” The number of ideal CVH factors was summed for each participant (range of 0–7 ideal characteristics) and this continuous variable was used as the primary predictor of interest during analysis.

### Measures of cognitive function

Five cognitive function assessments were used to create a summary variable of cognitive performance for each individual, with higher values indicating better cognitive function. These tests included immediate and delayed verbal recall, forward and backward digit span, and verbal fluency. During the immediate verbal recall test, interviewers read a list of 10 words aloud and asked the participants to immediately recall as many words as they could in 1 min. Three trials of this assessment were performed and the average test score was calculated. The interviewer then administered other cognitive tests, after which delayed recall ability was determined by asking subjects to remember the list of words without the interviewer reading the list again. The digit span test required participants to repeat back progressively longer series of numbers; the total score was recorded as the longest digit span repeated without error. This process was then repeated, but with the respondent repeating new sets of increasingly longer digit spans in reverse. The verbal fluency test consisted of naming as many animals as possible in 1 min; the final verbal fluency score was correct responses minus errors.

Preliminary analysis indicated that these tests were moderately correlated (Pearson correlation scores ranging from 0.327 to 0.706). Thus, principal component analysis was used to develop a composite cognitive performance from the battery of tests. This analysis computed component scores and weighted the contribution of each individual test to overall cognitive performance. The composite factor score resulting from this analysis (for each participant) was used as a measure of overall cognitive function during hypothesis testing.

### Participant characteristics

Individual characteristics known to influence cognitive performance were controlled for in all analyses. Evidence suggests that sex and age influence the extent of cognitive decline in older adults (21). Socio-economic factors appear to affect cognition, such that individuals with lower education and household income levels experience an increased risk of cognitive decline during aging (21). In addition, individuals living in rural settings have been shown to exhibit a higher dementia prevalence compared to urban areas (22). Reported annual household income was combined with an index of durable goods ownership, dwelling characteristics, and access to services; this wealth variable was then classified into household income quintiles for the analyses. Reported highest attained education level was standardized using the International Standard Classification of Education (23). Individual education level was then categorized as: (1) no formal schooling; (2) less than a high school degree; or, (3) high school degree or beyond.

### Statistical analyses

All analyses were conducted using Stata version 14, results were regarded as significant at *p* < 0.05. Household and individual weights were post-stratified according to country-specific population data (17). Linear regressions were conducted to test the hypothesis and evaluate the relative contribution of the total CVH score to composite cognitive performance score, while controlling for sociodemographic factors. Additional linear regressions were run to determine the associations between individual CVH components (categorized as ideal vs. not ideal) and composite cognitive performance. Individuals missing one or more variables were excluded from regression analysis (*n* = 2,071). Preliminary analysis indicated individuals missing data tended to be slightly older (mean of 62.1 years vs. 60.3 years), but otherwise there were no marked participant characteristic differences between individuals missing data and those included in the analysis. In addition, a single individual had zero CVH ideal traits, every other participant had at least one trait. This outlier disrupted the general linearity of the data and was there removed from analysis, resulting in a final sample of 11,295 participants.

## Results

### Descriptive statistics

Table 1 shows the characteristics of the study population (Table 1). The study population contained slightly more females (53.6%) than males (46.4%), but was roughly equally split between urban (50.6%) and rural (49.4%) households. Most participants had received some formal schooling, but had not earned a high school degree or the equivalent (59.0%). Over half the study participants reported ideal smoking (66.6%), drinking (92.6%), physical activity (60.5%), diet (87.1%), and anxiety levels (95.9%). Conversely, less than half of the subjects exhibited ideal BMI (38.9%) or BP (12.3%). Mean age was 63 years old and average number of combined ideal CVH measures was 4.5 (*SD* = 1.0). Cognitive test scores varied based on test type, from the wide range of scores evident on the verbal fluency test (mean of 12.74, range of 0–68) to the lower scores recorded on the backward digit span test (mean of 3.40, range of 0–8).

**Table 1:** Description of the Chinese study population (unweighted data), SAGE Wave 1 (2007–2010), *n* = 11,295 individuals.^a^^,^^b^.

**Variables**	***n* (%)**
Male sex	5,242 (46.4)
Urban dwelling	5,711 (50.6)
Income quintile 1 (low)	2,196 (19.4)
Income quintile 2	2,254 (20.0)
Income quintile 3	2,283 (20.2)
Income quintile 4	2,365 (20.9)
Income quintile 5 (high)	2,197 (19.5)
No formal education	2,558 (23.6)
Less than high school	6,660 (59.0)
High school or beyond	1,967 (17.4)
Ideal smoking	7,519 (66.6)
Ideal drinking	10,457 (92.6)
Ideal BMI	4,397 (38.9)
Ideal PAL	6,828 (60.5)
Ideal BP	1,384 (12.3)
Ideal diet	9,835 (87.1)
Ideal stress	10,835 (95.9)
**MEAN (RANGE)**	
Age	62.9 (50 – 95)
Combined ideals score	4.5 (−3.62 – 3.56)
Immediate verbal recall	5.62 (0 – 10)
Delayed verbal recall	4.97 (0 – 10)
Forward digit span	7.03 (0 – 9)
Backward digit span	3.40 (0 – 8)
Verbal fluency	12.74 (0 – 68)

a Body Mass Index (BMI), Blood Pressure (BP), Physical Activity Level (PAL)

b Ideal cardiovascular health measures:

Ideal smoking = never smoked

Ideal drinking = do not drink daily

Ideal BMI = 18.5–22.9 kg/m^2^

Ideal PAL ≥ 150 min/week

Ideal BP = ideal SBP (< 120 mm Hg) and ideal DBP (< 80 mm Hg)

Ideal diet = consuming ≥ 5 servings of fruit and/or vegetables daily combined with reporting access to an adequate amount of food

*Ideal stress = reporting either no or mild anxiety*.

### Combined CVH measures and composite cognitive function score

Total number of ideal CVH factors significantly contributed to variation in cognitive test results, although the association between total CVH traits and the composite cognitive score appears to be modest (Table 2). Still, a positive relationship was observed between number of CVH ideals and cognitive performance (B = 0.03, *P* < 0.05), indicating that a higher number of individual CVH traits corresponded to higher overall cognitive performance.

**Table 2:** Linear regression modeling the association between composite cognitive score and total number of CVH factors (ranging from 1 to 7 ideal traits) in the Chinese study population (weighted data), SAGE Wave 1 (2007–2010).

**Variable**	**β (SE)**
Sex	−0.106 (0.034)^**^
Age*:* 60–69 years old	−0.219 (0.026)^***^
70–79 years old	−0.498 (0.032)^***^
80+ years old	−0.931 (0.056)^***^
Income quintile: 1	−0.476 (0.066)^***^
2	−0.384 (0.060)^***^
3	−0.294 (0.062)^***^
4	−0.107 (0.050)^*^
Household setting	−0.215 (0.042)^***^
Education: < high school	0.484 (0.020)^***^
High school or beyond	0.872 (0.052)^***^
Total number of ideal CVH factors	0.031 (0.015)^*^

Parameter estimates (β) with standard error (SE).^a−c^

a *Cardiovascular Health (CVH*)

b Comparisons are statistically significant at:

* *P < 0.05*,

** *P < 0.01*,

*** P < 0.001

c Reference groups used in the creation of pacifier codes for each categorical variable:

Sex = 0 (male)

Age = 50-59 years old

Income quintile = 5 (high income)

Household setting = 0 (urban)

*Education level = no formal schooling*.

### Individual CVH measures and composite cognitive function score

Interestingly, regressions examining the individual contributions of ideal smoking, drinking, BMI, PAL, and BP to composite cognitive performance variation were not significantly related to the composite cognitive test scores (Table 3). Only ideal diet (B = 0.180, *P* < 0.001) and ideal anxiety (B = 0.308, *P* < 0.001) classifications were significantly associated with cognitive performance. As expected, ideal diet and ideal anxiety levels were related to higher overall cognitive performance.

**Table 3:** Linear regressions modeling associations between composite cognitive score (the dependent variable) and each individual CVH factor (the final independent variable entered in each model) in the Chinese study population (weighted data), SAGE Wave 1 (2007–2010).

	**Sex**	**Age**	***Income quintile*: 1**	***Income quintile*: 2**	***Income quintile*: 3**	***Income quintile*: 4**	**Household setting**	***Education*: < high school**	***Education*: high school or beyond**	**Ideal CVH trait**
Ideal Smoking	−0.092 (0.034)^**^	−0.029 (0.002)^***^	−0.460 (0.068)^***^	−0.369 (0.061)^***^	−0.280 (0.063)^***^	−0.098 (0.049)	−0.225 (0.043)^***^	0.472 (0.021)^***^	0.848 (0.052)^***^	0.006 (0.331)
Ideal Drinking	−0.095 (0.030)^**^	−0.029 (0.002)^***^	−0.462 (0.068)^***^	−0.371 (0.062)^***^	−0.278 (0.063)^***^	−0.098 (0.049)	−0.222 (0.043)^***^	0.472 (0.021)^***^	0.848 (0.052)^***^	0.038 (0.038)
Ideal BMI	−0.090 (0.028)^**^	−0.029 (0.002)^***^	−0.458 (0.067)^***^	−0.368 (0.061)^***^	−0.279 (0.063)^***^	−0.098 (0.049)	−0.224 (0.042)^***^	0.471 (0.021)^***^	0.849 (0.052)^***^	−0.018 (0.019)
Ideal PAL	−0.089 (0.028)^**^	−0.029 (0.002)^***^	−0.462 (0.065)^***^	−0.371 (0.058)^***^	−0.281 (0.060)^***^	−0.099 (0.047)^*^	−0.227 (0.044)^***^	0.472 (0.021)^***^	0.850 (0.052)^***^	0.012 (0.046)
Ideal BP	−0.089 (0.028)^**^	−0.029 (0.002)^***^	−0.461 (0.068)^***^	−0.370 (0.062)^***^	−0.280 (0.063)^***^	−0.098 (0.049)	−0.224 (0.043)^***^	0.472 (0.021)^***^	0.849 (0.052)^***^	0.021 (0.025)
Ideal Diet	−0.090 (0.028)^**^	−0.028 (0.002)^***^	−0.451 (0.068)^***^	−0.366 (0.062)^***^	−0.279 (0.063)^***^	−0.100 (0.049)^*^	−0.225 (0.043)^***^	0.467 (0.021)^***^	0.841 (0.053)^***^	0.130 (0.049)^*^
Ideal Anxiety	−0.087 (0.029)^**^	−0.028 (0.002)^***^	−0.451 (0.067)^***^	−0.362 (0.061)^***^	−0.277 (0.063)^***^	−0.096 (0.049)	−0.226 (0.042)^***^	0.469 (0.020)^***^	0.846 (0.052)^***^	0.242 (0.077)^**^

Full models including all covariates are presented; parameter estimates (β) with standard error (SE).^a−c^

a Body Mass Index (BMI), Blood Pressure (BP), Physical Activity Level (PAL)

b Comparisons are statistically significant at:

* *P < 0.05*,

** *P < 0.01*,

*** P < 0.001

c Reference groups used in the creation of pacifier codes for each categorical variable:

Sex = 0 (male)

Income quintile = 5 (high income)

Household setting = 0 (urban)

*Education level = no formal schooling*.

## Discussion

The results of this study generally support the hypothesis that ideal measures of the seven CVH factors are positively associated with cognitive performance on a battery of tests. Furthermore, our findings are consistent with the positive linear associations documented between ideal CVH metrics and mortality (24) and other non-cardiovascular outcomes (25) in meta-analyses.

### Overall CVH and cognitive performance

The total number of CVH ideal characteristics was positively related to composite cognitive performance score (Table 2). As expected, a higher number of ideal CVH traits corresponded with higher composite cognitive function scores, supporting previous findings in high-income nations (5, 6, 26), in addition to a meta-study of both US and non-US populations (27). Therefore, regardless of where implemented, a consistent pattern evident between a higher number of ideal CVH traits and lower prevalence of cognitive impairment—similar to what was found in our study. There are several potential explanations for the observed relationship between CVH and composite cognitive performance.

First, previous research indicates that improved cardiovascular health enhances vascular health and brain tissue perfusion, resulting in cognitive gains (28). High levels of general CVH therefore likely support learning and cognitive function later in life. Moreover, better vascular function also results from the avoidance of risky behaviors (like smoking and heavy drinking). These risky behaviors introduce toxins into the bloodstream, resulting in the development of atherosclerosis (a main contributor to cardiovascular disease) and causing systematic inflammation that impairs cognitive function (5). Thus, improved levels of cumulative CVH likely offer some protection from cognitive impairment during senescence.

### Individual CVH measures and cognitive performance

Unexpectedly, when the CVH measures were considered separately only ideal diet and anxiety were significantly associated with composite cognitive performance. These findings are likely due to the importance of proper nutrition to mental acuity and the detrimental effects of chronic anxiety. While fruit and vegetable intake is not a comprehensive measure of overall diet, it provides a macro-level indication of diet quality and key components of the diet in relation to disease burden. The association between produce consumption and cognitive function documented here is consistent with results from analyses of SAGE data conducted by other analysts. Specifically, previous work indicates that the consumption of both fruit and vegetables is related to higher cognitive health among SAGE participants (29). It should also be noted that a preliminary analysis testing for an interaction between BMI and diet in relation to cognitive function was not significant (*p* = 0.711), indicating that diet influences cognition independent of any relationship with BMI.

There are several possible mechanisms accounting for this association. For instance, it is possible that antioxidants in fruit and vegetables play an important role in preventing oxidative stress (e.g., caused by free radicals), thereby protecting neural cells from damage (30–32). Similarly, key nutrients found in produce (i.e., zinc) appear to directly protect neurological functions (33). Fruits and vegetables are also high in fiber, which may support a healthy gut microbiota linked with higher cognitive health (34). It should also be noted that a preliminary analysis testing for an interaction between BMI and diet in relation to cognitive function was not significant (*p* = 0.711), indicating that diet influences cognition independent of any relationship with BMI.

Likewise, the association between cognitive function and anxiety documented here may be explained by multiple factors. First, the energetic costs of chronic anxiety may contribute to this relationship. Chronic anxiety has long been known to affect the proper processing, storage, and mobilization of energetic reserves (i.e., glucose availability), which may have implications for energetically-expensive cognitive functions (35). Chronically elevated stress hormones have also been shown to alter brain cell structure and lead to a loss of neurons, impairing various aspects of cognition, such as memory (35). Moreover, personality traits (e.g., neuroticism) have been linked with self-reported anxiety and have also been connected to poor cognitive performance in older adults (36, 37). Aspects of participant personality may therefore also contribute to anxiety risk and subsequent cognitive decline.

The promotion of increased produce consumption and techniques to reduce anxiety levels may therefore represent promising strategies to support healthy cognitive function among older Chinese adults. This would also support findings in China which suggest that improvements in diet and physical activity are needed to improve CVH and prevent strokes (38). It is unknown why other CVH factors—identified in studies of other populations—did not significantly influence cognitive performance in this sample. We speculate that this may due to a combination of population characteristics and measurement techniques. For example, a relatively low percentage of participants in the study population were obese (14.3%); it is therefore possible that BMI does not have the same effect on cognitive performance as has been observed in wealthier nations with higher prevalence of obesity over the lifespan. Likewise, a large proportion of participants reported never having smoked (66.6%) or having consumed alcohol (69.1%) in their lifetimes. Again, this may decrease our ability to detect the influence of these behaviors on cognitive function.

The high rate of ideal CVH traits documented among the study population is consistent with recent work indicating that, compared to other populations outside of the United States, a larger proportion of Chinese adults exhibit 6–7 ideal CVH traits (15% of the study population; as defined using American Heart Association CVH measures) (27). Older Chinese adults may therefore generally exhibit higher levels of CVH, relative to older adults in other groups. Still, in concordance with the lack of significant associations between individual CVH traits and cognitive performance documented here, a review of how CVH interventions affect overall health in LMICs (including improvements in physical activity and body weight) found inconsistent patterns cross-culturally (39). This inconsistency suggests that the influence of individual CVH measures on health (including cognitive function) may vary across populations. Furthermore, the seven CVH factors used in the present study are interrelated; thus, a composite measure of overall CVH may function as a more reliable predictor for health outcomes.

### Limitations and strengths

The present study has several limitations. First, because the data are cross-sectional, it is not possible to determine causality between the variables assessed. It is therefore conceivable that cognitive impairment might cause poor CVH (due to changes in diet and activity patterns resulting from dementia). Second, while the battery of cognitive tests used to determine cognitive performance has been tested and used in numerous countries (6, 11, 14) and was tested during preliminary SAGE data collection, these cognitive measures were not developed in China. In the future, a more diverse range of cognitive tests may better capture different aspects of cognitive performance.

Furthermore, PALs were determined using self-report questionnaire-based data. Although this is a commonly used measure, self-report data often overestimates physical activity levels. Finally, this study was unable to use the exact ‘Life's Simple 7' CVH panel developed by the American Heart Association (6). Two of the variables included in this original panel (total cholesterol and fasting plasma glucose levels) were not collected as part of the SAGE Wave 1 study protocol. Two other factors known to influence CVH (drinking frequency and self-reported anxiety) were included in their place, potentially altering the comparability of results to similar analyses in other countries.

Despite these limitations, this study provides a unique examination of the associations between CVH and cognitive performance in older Chinese adults. Prior research testing these relationships has typically been limited to high-income countries and reliant on data collected from small and non-representative population samples. Conversely, the SAGE sample is large and captures the range of living conditions across China (16). Further, the use of self-reported anxiety levels in this study reveals the influence of mental health and perceived well-being on health outcomes, indicating this inexpensive measure may be a useful tool in future population risk assessments.

## Conclusion

These results support previous findings in high-income populations and suggest that general CVH is positively associated with cognitive test performance in older Chinese adults. Future research should therefore test whether increasing total number of patient CVH ideal traits is beneficial for cognitive function in an aging cohort. Decreasing the rate of neurological decline and promoting healthy cognitive aging will continue to be of utmost importance in coming years as the population of China continues to age and dementia rates increase. An increased focus on these topics is required to determine how targeting chronic disease risk factors (including poor CVH) can decrease physical and mental health burdens on older adult populations, thus contributing to improved health during aging.

## Data availability statement

The data that support the findings of this study are openly available in WHO Multi-Country Studies Data Archive at http://apps.who.int/healthinfo/systems/surveydata/index.php/catalog/13

## Author contributions

TG carried out background research, performed the statistical analysis, and drafted the manuscript. NN participated in designing the statistical methods used and helped to draft the manuscript. PK and FW conceived of the study and helped to draft the manuscript. YG and JS participated in the study design and helped draft the manuscript. All authors read and approved the final manuscript.

### Conflict of interest statement

The authors declare that the research was conducted in the absence of any commercial or financial relationships that could be construed as a potential conflict of interest.
